# Multicenter epidemiological distribution, pathogens, and drug-resistance characteristics of chronic osteomyelitis in Central China

**DOI:** 10.3389/fpubh.2025.1654861

**Published:** 2025-08-29

**Authors:** Zemin Liu, Guochao Jin, Le Zhang, Ruqi Zhang, Dong Wang, Min Liu, Yan Li, Yonghong Zhang

**Affiliations:** ^1^Second Clinical Medical College, Shanxi Medical University, Taiyuan, China; ^2^Department of Orthopaedics, Second Hospital of Shanxi Medical University, Taiyuan, China; ^3^School of Public Health, Shanxi Medical University, Taiyuan, China; ^4^Lucheng District Health Development Center, Changzhi, China; ^5^Wenxi County Center for Disease Prevention and Control, Yuncheng, China

**Keywords:** chronic osteomyelitis, multicenter, epidemiology, bacterial culture, antibiotic resistance, risk factors

## Abstract

**Introduction:**

The incidence of chronic osteomyelitis increases annually. Currently, epidemiological data on chronic osteomyelitis in Central China are scarce. Describing the epidemiological distribution, pathogens, and drug resistance characteristics of patients with chronic osteomyelitis in Central China is critical for its prevention and control. In this study, we aimed to statistically analyze patients with chronic osteomyelitis from four local hospitals to provide a reference for prevention and control in this region.

**Methods:**

We conducted a retrospective analysis of the clinical data of patients with chronic osteomyelitis admitted to four hospitals in Central China between January 1, 2016, and December 31, 2021, using ICD codes in the electronic medical record system. Data extracted included patients’ basic details, hospitalization records, infection status, and bacterial culture results. Statistical descriptions included pathogen detection, distribution, and changes. Furthermore, we analyzed the antibiotic resistance of Gram-positive and -negative bacteria. Patients with positive cultures underwent risk-factor analysis.

**Results:**

We included 632 patients with chronic osteomyelitis, comprising traumatic osteomyelitis (464 patients), hematogenous osteomyelitis (120 patients), and diabetic foot osteomyelitis (48 patients). The median age was 46 (IQR 30–58) years, and the majority were aged between 41 and 60 years. The male-to-female ratio was 2.49:1. Traffic accidents were identified as the primary etiological factor. The three most commonly affected sites were the tibia, femur, and calcaneus. Among the laboratory indicators, the erythrocyte sedimentation rate had the highest positive rate at 57.71%. Bacterial cultures were performed on the intraoperative specimens of 421 patients and yielded 292 positives (69.36%), of which we identified 386 isolates from 74 pathogens. The most prevalent pathogen were *Staphylococcus aureus*, *Coagulase-negative staphylococci*, and *Enterobacter* spp. (132 [34.20%], 47 [12.18%], and 25 [6.47%] isolates). Gram-positive bacteria were resistant to conventional antibiotics, including penicillin, erythromycin, and clindamycin; however, they were susceptible to peptide antibiotics and oxazolidinones. Gram-negative bacteria were resistant to first- and second-generation cephalosporins; nevertheless, they were sensitive to third- and fourth-generation cephalosporins as well as combinations such as cefoperazone-sulbactam. Multivariate logistic regression analysis identified length of hospital stay (odds ratio [OR] = 1.037, *p* = 0.001), diabetes (OR = 6.61, *p* = 0.049), and smoking (OR = 6.873, *p* = 0.003) as independent risk factors.

**Conclusion:**

Chronic osteomyelitis predominantly affects middle-aged and older adults, males, and those with tibial injuries from traffic accidents requiring special attention. *Staphylococcus aureus* was associated with the highest risk of infection. Empirical early-phase medication should be followed by targeted antibiotic therapy based on bacterial sensitivity and resistance. Patients with prolonged hospitalization, diabetes, or a smoking history require special attention. Notably, strengthening health education and postoperative care in these patients can reduce the risk of chronic osteomyelitis.

## 1 Introduction

Chronic osteomyelitis is a persistent inflammatory process that causes bone destruction following bacterial infection (1). Previously, it was caused by hematogenous osteomyelitis; however, it is currently caused by improper treatment of post-traumatic or post-fracture surgery infections or by limb infections secondary to diabetes (2, 3). Chronic osteomyelitis causes severe pain and functional impairment, markedly reducing quality of life. In addition, it leads to severe complications such as fracture non-union, limb deformities, and amputation. This imposes a heavy psychological and economic burden on patients (3–5). There’s a lack of epidemiological data on the incidence of chronic osteomyelitis in adults from low-income countries. The incidence of childhood chronic osteomyelitis in these countries is 43–200 cases per 100,000 people due to limited economic resources, medical services, and health awareness. This is far exceeding the 7.8–9.1 cases per 100,000 in high-income countries (6, 7). The incidence of chronic osteomyelitis has continues to rise. The incidence of adult chronic osteomyelitis in Germany increased by 10.44% in 2018 compared with 2008 (8). Furthermore, the prevalence of chronic osteomyelitis in the US grew from 11.4 cases per 100,000 people in 1969–1979 to 24.4 cases per 100,000 from 2000 to 2009 (9).

The incidence of osteomyelitis caused by traumatic factors, such as traffic accidents, is increasing in tandem with China’s economic growth. Ren et al. (10) reported that the incidence of post-traumatic limb-related osteomyelitis in China increased from 0.93% in 2008 to 2.16% in 2017. Notably, the rate decreased from 1.72–0.49% in Eastern China, whereas other regions with slower economic development saw a significant increase. Chronic osteomyelitis, with its long course and tendency to relapse, greatly impacts patients’ lives and work, necessitating focused attention. Antibiotics are used widely for its treatment; however, their improper use has led to increased antibiotic resistance (11). Therefore, clarifying local antibiotic resistance patterns in chronic osteomyelitis treatment is crucial for guiding local management. Recently, epidemiological studies on chronic osteomyelitis in northern, southern, southwestern, and northwestern China have guided local prevention and control (12–17). However, studies on the epidemiological distribution, pathogens, and resistance characteristics of chronic osteomyelitis are lacking in China’s central Loess Plateau region, where there is slow economic development and numerous patients with chronic osteomyelitis. In this study, we conducted a multicentre retrospective analysis of patients with chronic osteomyelitis from four local hospitals to delineate for prevention and control in this region.

## 2 Materials and methods

### 2.1 Data collection

We retrospectively analyzed the medical records of 632 patients diagnosed with ICD-10-coded (M86) diagnosis of chronic osteomyelitis admitted between January 1, 2016, and December 31, 2021. The patients were recruited from four hospitals, including the Second Hospital of Shanxi Medical University, Shanxi Provincial People’s Hospital, Lingfen People’s Hospital, and Yuncheng Central Hospital. We included only the first admission data for patients with multiple admissions. The study was approved by the institutional review boards of all participating hospitals.

### 2.2 Inclusion and exclusion criteria

The inclusion criteria were as follows: (1) Patients who have chronic osteomyelitis with clinical symptoms and signs, including pain, swelling, and sinus tract formation at the affected site, lasting for at least 4 weeks. Imaging studies (radiography, CT, MRI, etc.) involving the characteristic features of chronic osteomyelitis, including bone destruction, periosteal reactions, and sequestrum formation. (2) Complete clinical data, comprising medical history, examination reports, and treatment records, to facilitate comprehensive assessment and treatment outcomes for chronic osteomyelitis.

Exclusion Criteria: (1) Patients with acute osteomyelitis, defined by a short duration and features such as sudden high-grade fever, bone pain, and localized redness, swelling, warmth, and tenderness, without evolution to chronic osteomyelitis. (2) Patients with concomitant severe diseases, including those affecting major organs or with hematological disorders or immunodeficiencies, that could alter the disease progression and treatment efficacy of chronic osteomyelitis or study interpretation.

### 2.3 Pathogen detection

Intra-operative specimens were collected from 421 patients and comprised inflamed soft tissue, affected bone, biofilm on any implants, and/or pus at the infection site. All samples were transported to the central laboratory within 2 h for bacterial culture and antimicrobial susceptibility testing. Regarding pathogen collection, the Clinical and Laboratory Standards Institute recommended disk diffusion or automated instrumentation methods for isolating and culturing pathogens from intraoperative bone infection tissue samples and to conduct antibiotic susceptibility and resistance tests (Antimicrobial susceptibility was interpreted according to CLSI breakpoints). An organism was considered the causative agent when isolated from ≥ 3 identical samples. Growth was recorded when ≥ 2 samples per patient were positive. Multidrug resistance (MDR) was defined, following Magiorakos et al. (18), as acquired non-susceptibility to at least one agent in three or more antimicrobial categories.

### 2.4 Observation indicators

The indices included patient age, sex, admission year, type of chronic osteomyelitis, injury etiology, infection site, laterality, length of hospital stay, disease duration, body mass index (BMI), smoking history, alcohol history, trauma history, comorbidities, pathogen species, antibiotic susceptibility results, and laboratory markers including white blood cell count (WBC), erythrocyte sedimentation rate (ESR), and C-reactive protein (CRP).

We restricted the analysis to hospitalizations initiated specifically for the diagnosis and treatment of osteomyelitis. Disease duration was calculated as the interval (in months) from the first documented symptom attributable to osteomyelitis to the date on which chronic osteomyelitis was diagnosed by the treating physician based on imaging (MRI or CT) and/or positive intra-operative cultures.

All biochemical tests were performed in the central laboratories of the participating hospitals using certified analyzers; the authors conducted no wet-lab assays. All data were retrieved by the authors from the hospitals’ information systems and subsequently analyzed.

### 2.5 Statistical analysis

Statistical analyses were performed using the GraphPad Prism 10 software (GraphPad Software, San Diego, CA, United States). Normally distributed continuous data are expressed as mean ± standard deviation, and skewed data as median [interquartile range (IQR)]. Categorical data are presented as n (%). For the group comparisons, normally distributed data were analyzed using t-tests or one way analysis of variance, and non-normally distributed data were analyzed using the Mann–Whitney U or Kruskal–Wallis tests. Categorical data were compared using the chi-square test. The binary logistic regression model included variables significantly associated with chronic osteomyelitis in univariate analysis. Statistical significance was set at *p* < 0.05.

## 3 Results

### 3.1 General data

#### 3.1.1 Patient characteristics

A total of 632 patients were enrolled: 451 males and 181 females, with a male-to-female ratio of 2.49:1. The median age of the patients was 46 (IQR 30–58) years. Patients aged 41–60 years constituted the largest subgroup (249 patients, 39.4% of the total). Patients aged ≤ 20 years had the smallest number, 100 (15.82%). Following age stratification, the highest male-to-female ratio of 3.18:1 was observed in the 21–40 year age group, whereas the lowest (1.5:1) was observed in patients < 20 years old. The median length of hospital stay was 17 (IQR 11–26) days, and the median disease duration was 5 (IQR 2–16) months. The median BMI was 24.05 (IQR 21.81–25.39) kg/m^2^. Among the 632 patients with chronic osteomyelitis, 226 (35.8%) had a history of smoking, 148 patients (23.4%) reported alcohol consumption, 418 patients (66.1%) had undergone surgery, 350 patients (55.4%) had experienced trauma, and 209 patients (33.1%) had comorbidities. Specifically, 83 and 71 patients (13.13 and 11.23%) had a history of diabetes and hypertension.

Among the 632 patients, 464 patients (73.42%) had traumatic osteomyelitis (TO), 120 patients (18.99%) had hematogenous osteomyelitis (HO), and 48 patients (7.59%) had diabetic osteomyelitis (DO). Patients with DO were older, with a median age of 58 (IQR 48–67.75) years. In contrast, patients with HO were younger, with a median age of 35.5 (IQR 13.75–49) years. TO and DO peaked in the 21–40 years, whereas HO was most prevalent in patients aged < 20 years. TO had the highest male-to-female ratio of 2.90:1, whereas DO had the lowest male-to-female ratio of 1.67:1. Patients with HO had the shortest median hospital stay of 14 (IQR 9–23) days, while patients with DO had the longest at 20 (IQR 12.5–29.25) days. Patients with TO had the longest disease duration, with a median of 6 (IQR 2–22.50) months, compared with patients with DO within the shortest duration of 2 (IQR 1–8) months (Table 1).

**Table 1:** Basic characteristics of 632 patients with chronic osteomyelitis.

Items	Total	TO	HO	DO	*P*–value
Age [years, median (IQR)]	46 (IQR 30–58)	47 (IQR 32–59)	35.5 (IQR 13.75–49)	58 (IQR 48–67.75)	<0.001
Age [*n* (%)]					
≤20	100 (15.82)	59 (12.72)	40 (33.33)	1 (2.08)	–
21–40	138 (21.84)	106 (22.84)	27 (22.50)	5 (10.42)	–
41–60	249 (39.40)	187 (40.30)	38 (31.67)	24 (50)	–
≥61	145 (22.94)	112 (24.14)	15 (12.50)	18 (37.50)	–
Sex ratio (Males/Females)	451/181	345/119	76/44	30/18	0.021
Body laterality (Left/Right/Bilateral)	320/300/12	241/214/9	55/62/3	24/24/0	0.701
BMI [kg/m^2^, median (IQR)]	24.05 (IQR 21.81–25.39)	24.22 (IQR 22.04–25.57)	23.80 (IQR 19.74–25.35)	23.96 (IQR 22.41–24.66)	0.086
Hospitalization time [days, median (IQR)]	17 (IQR 11–26)	17 (IQR 12–26)	14 (IQR 9–23)	20 (IQR 12.5–29.25)	0.003
Duration of illness [months, median (IQR)]	5 (IQR 2–16)	6 (IQR 2–22.50)	5 (IQR 2–12)	2 (IQR 1–8)	0.002
Serum levels of preoperative inflammation markers					
WBC [×109/L, median (IQR)]	6.5 (IQR 5.42–8.01)	6.5 (IQR 5.49–7.93)	6.5 (IQR 5.20–8.14)	6.6 (IQR 4.80–8.7)	0.962
ESR [mm/h, median (IQR)]	20.5 (IQR 10–47.50)	20 (IQR 8–40)	20 (IQR 10–51.25)	60 (IQR 20–70)	<0.001
CRP [mg/L, median (IQR)]	6.87 (IQR 3.14–19)	6.82 (IQR 3.14–18.13)	5.38 (IQR 2.70–17.48)	13.80 (IQR 3.78–47.78)	0.078
Positive rates of preoperative inflammation markers (%)					
WBC	12.50	10.34	4.53	20.83	0.019
ESR	40.03	38.14	40	58.33	0.027
CRP	32.28	31.90	28.33	45.83	0.089

#### 3.1.2 Types and causes of chronic osteomyelitis

Of the 632 patients, 286 patients (45.25%) and 181 patients (28.64%) had chronic osteomyelitis caused by open injuries and closed injuries, whereas 165 patients (26.11%) were non-trauma-induced. The top four causes of injury were traffic accidents 176 patients (27.85%), falls 89 patients (14.08%), crush injuries 41 patients (6.49%), and struck-by-object injuries 38 patients (6.01%).

#### 3.1.3 Infection laterality and site

**Figure 1:**
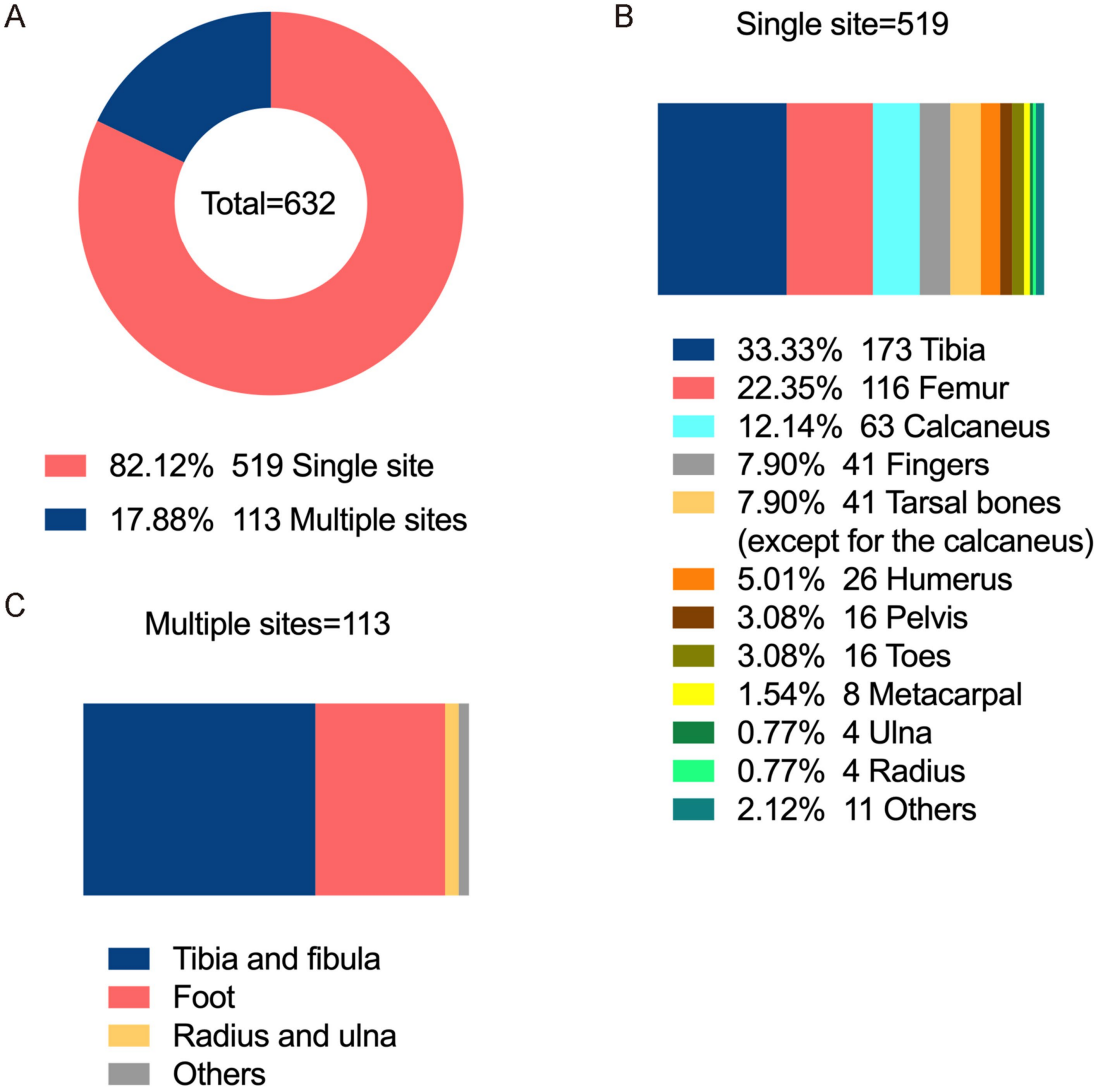
Infection sites of chronic osteomyelitis. **(A)** Distribution of single-site and multi-site limb infections. **(B)** Proportion of single-site limb infections. **(C)** Proportion of multi-site limb infections.

Regarding laterality, chronic osteomyelitis was left-sided in 320 patients (50.63%), right-sided in 300 patients (47.47%), and bilateral in 12 patients (1.90%). The infection predominantly affected the lower and upper limbs and the trunk bones in 517 patients (81.80%), 92 patients (14.56%), and 23 patients (3.64%). Single-site infection occurred 519 patients (82.12%) whereas multiple-site infection was observed 113 patients (17.88%). The top three sites for single-site infections were the tibia 173 patients (33.33%), femur 116 patients (22.35%), and calcaneus 63 patients (12.14%). Among multiple-site infections, the most common were tibial-fibular involvement 68 patients (60.18%), followed by foot infections 38 patients (33.63%) (Figure 1).

### 3.2 Serum inflammatory marker levels and positivity rates for chronic osteomyelitis

The normal values for serum inflammatory markers that were provided by the clinical laboratory are as follows: WBC: 4–10 × 10^9^/L, ESR: 15 and 20 mm/h for men and women, and CRP: 8 mg/L. Patients with chronic osteomyelitis had WBC, CRP, and ESR levels of 6.5 (IQR 5.42–8.01) × 10^9^/L, 6.87 (IQR 3.14–19) mg/L, and 20.5 (IQR 10–47.5) mm/h. The positivity rates were 12.5, 32.28, and 40.03% for WBC, CRP, and ESR. DO showed the highest positivity rates (20.83, 45.83, and 58.33% for WBC, CRP, and ESR) (Table 1).

### 3.3 Characteristics of pathogen infection

#### 3.3.1 Pathogen detection rate

Surgical specimens were collected from 421 of 632 patients with chronic osteomyelitis. Of these, 292 patients were positive using the bacterial culture (positivity rate, 69.36%). We identified 386 isolates from 74 pathogens: Gram-positive bacteria 244 strains (63.21%), Gram-negative bacteria 139 strains (36.01%), and fungi 3 strains (0.78%). The overall pathogen and multidrug-resistant bacterial detection rate was 91.68% (386/421) and 44.04% (170/386), respectively. Table 2 shows the annual pathogen detection rates. Overall, the positivity rates for microbial culture, pathogen detection, and multidrug-resistant bacteria detection fluctuated slightly; however, it showed a general upward trend over the years.

**Table 2:** Antimicrobial resistance trends of *methicillin-sensitive Staphylococcus aureus* to key antimicrobial agents.

Antibacterial drugs	2016 *n* = 14	2017 *n* = 15	2018 *n* = 12	2019 *n* = 15	2020 *n* = 26	2021 *n* = 19	Total *n* = 101
Ciprofloxacin	7.14	16.67	0.00	0.00	5.00	7.14	6.49
Levofloxacin	0.00	11.11	0.00	0.00	7.69	5.88	4.76
Moxifloxacin	0.00	0.00	0.00	0.00	7.69	0.00	2.44
Gentamycin	7.14	26.67	25.00	20.00	4.00	15.79	15.00
Erythromycin	53.85	66.67	58.33	60.00	50.00	57.89	57.00
Penicillin	100.00	100.00	83.33	100.00	100.00	100.00	98.00
Ampicillin	ND*	ND*	ND*	ND*	ND*	50.00	50.00
Amoxicillin-Clavulanate	25.00	0.00	0.00	0.00	ND*	ND*	6.90
Tetracycline	14.29	14.29	16.67	6.67	3.85	5.56	9.09
Rifampicin	7.69	0.00	0.00	6.67	0.00	0.00	2.00
Clindamycin	35.71	60.00	58.33	53.33	48.00	50.00	50.51
Cotrimoxazole	0.00	33.33	25.00	7.14	11.54	16.67	15.31
Vancomycin	8.33	0.00	0.00	0.00	0.00	0.00	1.03

*ND indicates that antimicrobial susceptibility testing for these agents was not performed.

#### 3.3.2 Pathogen distribution

**Figure 2:**
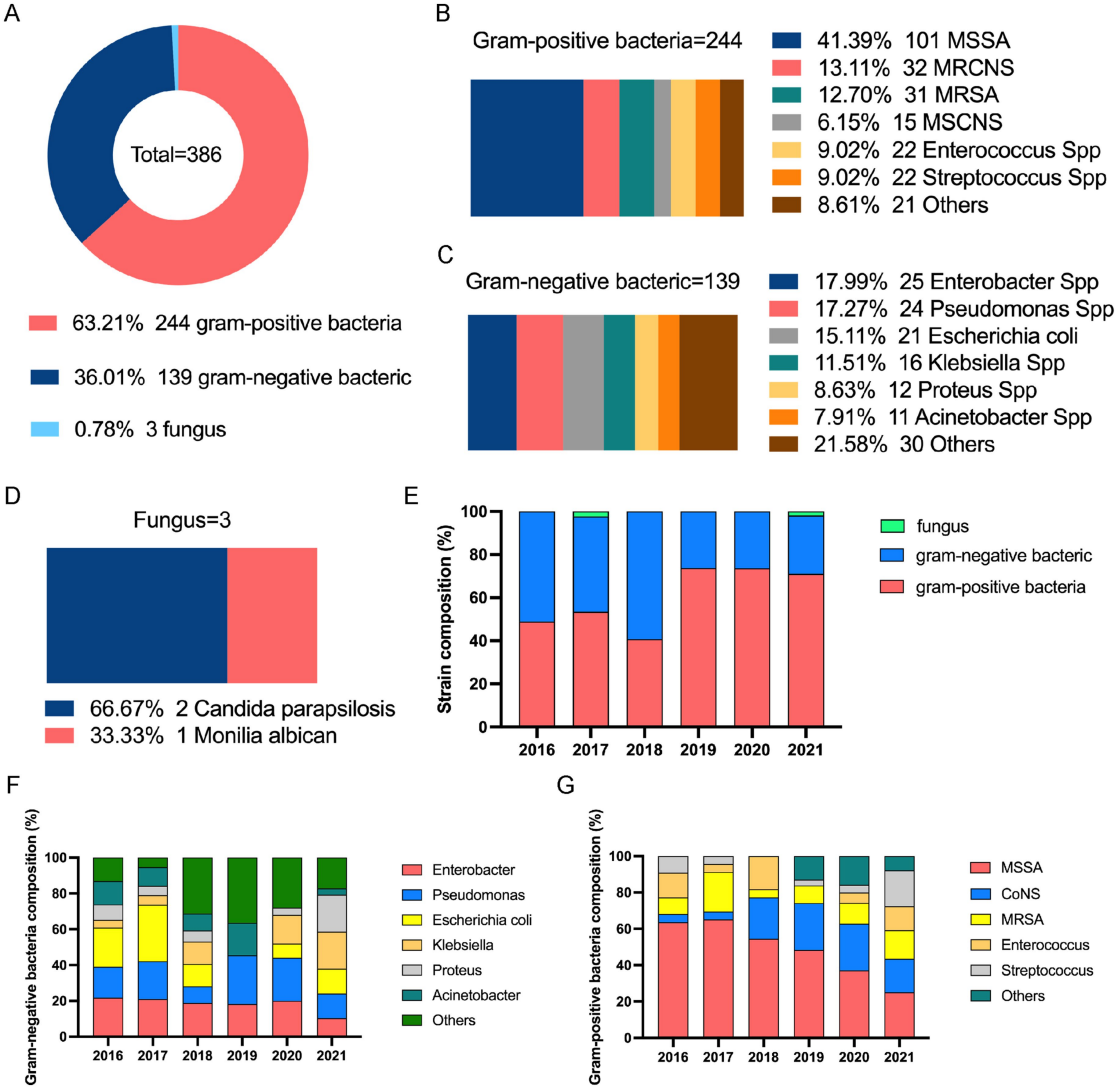
Distribution of pathogen cultures in patients with chronic osteomyelitis (2016–2021). **(A)** Proportion of infections by pathogen type. **(B)** Proportion of Gram-positive bacterial infections. **(C)** Proportion of Gram-negative bacterial infections. **(D)** Proportion of fungal infections. **(E)** Trend in proportion of infections by pathogen type over time. **(F)** Proportion trend of Gram-positive bacterial infections over time. **(G)** Proportion trend of Gram-negative bacterial infections over time. MSCNS, *methicillin-sensitive coagulase-negative staphylococci*; MRSNS, *methicillin-resistant coagulase-negative staphylococci*; MSSA, *methicillin-se*nsitive *Staphylococcus aureus*; MRSA, *methicillin-resistant Staphylococcus aureus*; CoNS, *coagulase-negative staphylococci*.

Among the 386 isolates, *Staphylococcus aureus* (*S. aureus*) was the most prevalent 132 strains (34.20%), followed by *Coagulase-negative staphylococci* (CoNS) 47 strain (12.18%), *Enterobacter* spp. 25 strains (6.47%), *Pseudomonas* spp. 244 strains (6.22%), *Enterococcus* spp. 22 strains (5.70%), *Streptococcus* spp. 22 strains (5.70%), *Escherichia coli* 21 strains (5.44%), and others 93 strains (24.09%) (Figure 2).

Among Gram-positive bacteria, *S. aureus* had the highest rate: 132 strains (132/244, 54.10%), comprising 31 *methicillin-resistant S. aureus* (MRSA) strains and 101 *methicillin-sensitive S. aureus* (MSSA) strains. This was followed by 47 CoNS strains (47/244, 19.26%), including 32 *methicillin-resistant coagulase-negative staphylococci* (MRCNS) and 15 *methicillin-sensitive coagulase-negative staphylococci* (MSCNS) strains; 22 *Enterococcus* strains (22/244, 9.02%); and 22 *Streptococcus strains* (22/244, 9.02%) (Figure 2).

Among the Gram-negative bacteria, *Enterobacter* species were detected (25/139, 17.99%), followed by *Pseudomonas* spp. 24 strains (17.27%), *Escherichia coli* 21 strains (15.11%), *Klebsiella* species (16, 11.51%), *Proteus* species 12 strains (8.63%), and *Acinetobacter* species 11 strains (7.91%). We discovered mixed infections with two or more bacterial species in 79 cases (79/292, 27.05%). The distribution of Gram-positive and -negative bacteria in single versus mixed infections differed significantly (chi-square = 8.688, *p* = 0.013).

### 3.4 Antimicrobial resistance of major Gram-positive bacteria

#### 3.4.1 Antimicrobial resistance of MSSA

The MSSA showed varying degrees of resistance to most antimicrobials. The highest rate (98%) was against penicillin and >50% for erythromycin, lincosamides, and ampicillin. Susceptibility was maintained to quinolones, rifamycins, tetracyclines, and peptides, with resistance below 10%. Gentamicin and cotrimoxazole resistance ranged from 15 to 20%. Between 2016 and 2021, high resistance to penicillin, erythromycin and clindamycin remained stable. Gentamicin resistance increased from 7.14–15.79%, whereas tetracycline resistance fell from 14.29–5.56% (Table 2).

#### 3.4.2 Antimicrobial resistance of MRSA

MRSA showed high resistance to all antibiotics except vancomycin, with 100% resistance to amikacin, penicillin, and ampicillin. Erythromycin, amoxicillin-clavulanate, and clindamycin resistance exceeded 70%. Fluoroquinolone resistance ranged from 20.69–31.03%, and resistance gentamicin and tetracycline resistance exceeded 30%. Rifampicin and cotrimoxazole resistance exceeded 10% (Table 3).

**Table 3:** Antimicrobial resistance trends of *methicillin-resistance Staphylococcus aureus* to key antimicrobial agents.

Antibacterial drugs	2016 *n* = 2	2017 *n* = 5	2018 *n* = 1	2019 *n* = 3	2020 *n* = 8	2021 *n* = 12	Total *n* = 31
Ciprofloxacin	50.00	50.00	0.00	0.00	50.00	16.67	27.27
Levofloxacin	50.00	0.00	0.00	100.00	37.50	27.27	31.03
Moxifloxacin	0.00	0.00	0.00	100.00	37.50	18.18	20.69
Gentamycin	50.00	50.00	0.00	100.00	37.50	16.67	30.00
Amikacin	ND*	ND*	ND*	ND*	ND*	100.00	100.00
Erythromycin	100.00	100.00	100.00	100.00	62.50	91.67	86.67
Oxacillin	100.00	100.00	100.00	100.00	100.00	100.00	100.00
Penicillin	100.00	100.00	100.00	100.00	100.00	100.00	100.00
Ampicillin	ND*	ND*	ND*	ND*	ND*	100.00	100.00
Amoxicillin-Clavulanate	ND*	100.00	ND*	50.00	ND*	100.00	75.00
Tetracycline	100.00	20.00	0.00	100.00	25.00	33.33	36.67
Rifampicin	0.00	0.00	0.00	100.00	0.00	8.33	10.34
Clindamycin	100.00	100.00	100.00	100.00	37.50	75.00	74.19
Cotrimoxazole	0.00	20.00	0.00	0.00	33.33	8.33	13.79
Vancomycin	0.00	0.00	0.00	0.00	0.00	8.33	3.33

*ND indicates that antimicrobial susceptibility testing for these agents was not performed.

#### 3.4.3 Antimicrobial resistance of CoNS

The CoNS displayed >50% resistance to penicillin, erythromycin, amoxicillin-clavulanate, and fosfomycin. Quinolones, tetracyclines, aminoglycosides, lincosamides, and sulphonamides resistance was from 10–50%. Rifampicin, quinupristine, dalfopristin, teicoplanin, linezolid, and vancomycin resistance was <10% (Table 4).

**Table 4:** Antimicrobial resistance trends of *coagulase-negative staphylococci* to key antimicrobial agents.

Antibacterial drugs	2016 *n* = 1	2017 *n* = 1	2018 *n* = 5	2019 *n* = 8	2020 *n* = 18	2021 *n* = 14	Total *n* = 47
Ciprofloxacin	0.00	100.00	25.00	0.00	25.00	25.00	26.32
Levofloxacin	0.00	100.00	40.00	25.00	27.78	35.71	31.91
Moxifloxacin	0.00	100.00	0.00	33.33	18.75	21.43	20.51
Gentamycin	0.00	100.00	40.00	16.67	18.75	28.57	25.58
Erythromycin	100.00	100.00	100.00	62.50	61.11	42.86	61.70
Oxacillin	100.00	100.00	40.00	75.00	66.67	71.43	68.09
Penicillin	100.00	100.00	100.00	83.33	87.50	78.57	86.05
Amoxicillin-Clavulanate	ND*	ND*	100.00	0.00	ND*	ND*	50.00
Tetracycline	0.00	0.00	0.00	33.33	18.75	23.08	19.51
Rifampicin	0.00	0.00	0.00	20.00	0.00	7.69	8.33
Clindamycin	100.00	0.00	60.00	37.50	44.44	50.00	48.94
Cotrimoxazole	0.00	0.00	100.00	42.86	33.33	50.00	45.65
Quinupristin-Dalfoprisdn	0.00	ND*	0.00	ND*	0.00	12.50	5.00
Linezolid	0.00	0.00	0.00	0.00	0.00	14.29	4.26
Vancomycin	0.00	0.00	0.00	0.00	0.00	0.00	0.00
Teicoplanin	ND*	0.00	100.00	0.00	0.00	0.00	3.23
Fosfomycin	ND*	ND*	ND*	50.00	ND*	ND*	50.00

*ND indicates that antimicrobial susceptibility testing for these agents was not performed.

### 3.5 Antimicrobial resistance of Gram-negative bacteria

#### 3.5.1 Antimicrobial resistance of *Enterobacter* species

The *Enterobacter* species showed varying degrees of resistance to all tested antimicrobials. Resistance to first- and second-generation cephalosporins was higher than that to the third- and fourth-generation, with lower resistance to cephalosporin/enzyme inhibitor combinations. Ciprofloxacin, gentamycin, tobramycin, piperacillin, and aztreonam resistance was between 10 and 30%. In contrast, resistance to ampicillin and ampicillin/sulbactam was 100%. Levofloxacin, amikacin, and piperacillin-tazobactam resistance was <10% (Table 5).

**Table 5:** Antimicrobial resistance trends of *Enterobacter* species to key antimicrobial agents.

Antibacterial drugs	2016 *n* = 5	2017 *n* = 4	2018 *n* = 6	2019 *n* = 2	2020 *n* = 5	2021 *n* = 3	Total *n* = 25
Ciprofloxacin	40.00	25.00	0.00	0.00	0.00	50.00	17.39
Levofloxacin	25.00	25.00	0.00	0.00	0.00	0.00	9.09
Amikacin	0.00	0.00	16.67	0.00	0.00	33.33	8.00
Gentamycin	50.00	0.00	0.00	0.00	0.00	100.00	21.05
Tobramycin	0.00	0.00	20.00	0.00	0.00	50.00	11.76
Ampicillin	100.00	ND*	ND*	ND*	100.00	ND*	100.00
Ampicillin-Sulbactam	100.00	ND*	ND*	ND*	100.00	ND*	100.00
Piperacillin	ND*	0.00	20.00	100.00	20.00	100.00	26.67
Piperacillin-Tazobactam	25.00	0.00	0.00	0.00	0.00	0.00	4.17
Amoxicillin-Clavulanate	ND*	0.00	33.33	0.00	100.00	100.00	55.56
Aztreonam	50.00	0.00	16.67	50.00	0.00	66.67	25.00
Cefazolin	100.00	100.00	33.33	0.00	100.00	100.00	86.67
Cefuroxime	100.00	0.00	50.00	0.00	66.67	100.00	66.67
Cefotetan	100.00	ND*	33.33	ND*	66.67	ND*	62.50
Cefoxitin	100.00	ND*	33.33	100.00	100.00	100.00	80.00
Ceftriaxone	50.00	0.00	16.67	50.00	20.00	66.67	28.57
Cefoperazone-Sulbactam	33.33	0.00	0.00	0.00	0.00	0.00	7.14
Cefotaxime	100.00	0.00	25.00	ND*	ND*	ND*	40.00
Ceftazidime	100.00	0.00	20.00	50.00	20.00	33.33	31.82
Cefepime	20.00	0.00	0.00	0.00	0.00	33.33	8.33

*ND indicates that antimicrobial susceptibility testing for these agents was not performed.

#### 3.5.2 Antimicrobial resistance of *Pseudomonas* species

*Pseudomonas* spp. demonstrated lower resistance to third-and fourth-generation cephalosporins than to first-and second-generation cephalosporins. The resistance to ciprofloxacin, amikacin, gentamycin, and meropenem was low (<15%). However, resistance was 40–100% for ticarcillin-clavulanic acid, and ampicillin, whereas it was below 25% for other β-lactam antibiotics (Table 6).

**Table 6:** Antimicrobial resistance trends of *Pseudomonas* species to key antimicrobial agents.

Antibacterial drugs	2016 *n* = 4	2017 *n* = 4	2018 *n* = 3	2019 *n* = 3	2020 *n* = 6	2021 *n* = 4	Total *n* = 24
Ciprofloxacin	0.00	25.00	0.00	0.00	16.67	0.00	8.33
Levofloxacin	25.00	25.00	0.00	0.00	16.67	0.00	12.50
Amikacin	25.00	0.00	0.00	0.00	0.00	0.00	4.17
Gentamycin	0.00	25.00	0.00	0.00	20.00	0.00	9.52
Tobramycin	50.00	25.00	0.00	0.00	0.00	0.00	13.04
Piperacillin	25.00	25.00	0.00	0.00	16.67	0.00	13.04
Piperacillin-Tazobactam	25.00	0.00	0.00	0.00	20.00	0.00	9.09
Ampicillin	0.00	ND*	100.00	ND*	ND*	ND*	40.00
Ampicillin-Sulbactam	0.00	ND*	100.00	ND*	ND*	ND*	25.00
Ticarcillin-Clavulanate	100.00	0.00	ND*	ND*	0.00	66.67	57.14
Imipenem	0.00	25.00	0.00	0.00	0.00	0.00	4.35
Meropenem	0.00	25.00	0.00	0.00	0.00	0.00	4.35
Aztreonam	0.00	0.00	0.00	0.00	50.00	0.00	15.79
Cefazolin	50.00	ND*	100.00	ND*	100.00	ND*	85.71
Cefuroxime	100.00	ND*	100.00	ND*	ND*	ND*	100.00
Cefotetan	0.00	ND*	100.00	ND*	ND*	ND*	25.00
Cefoxitin	ND*	ND*	100.00	ND*	ND*	ND*	100.00
Ceftazidime	0.00	0.00	0.00	0.00	16.67	0.00	4.35
Ceftriaxone	0.00	ND*	100.00	ND*	ND*	ND*	40.00
Cefoperazone-Sulbactam	100.00	0.00	0.00	0.00	16.67	0.00	11.76
Cefepime	0.00	0.00	0.00	0.00	16.67	0.00	4.17
Cotrimoxazole	50.00	ND*	100.00	ND*	ND*	ND*	75.00
Tigecycline	ND*	ND*	100.00	ND*	ND*	ND*	100.00

*ND indicates that antimicrobial susceptibility testing for these agents was not performed.

#### 3.5.3 Antimicrobial resistance of *Escherichia coli*

*Escherichia coli* demonstrated >60% resistance to first-generation cephalosporins, cefazolin, and second-generation, cefuroxime. In addition, over 50% resistance to cefotaxime (third-generation) and <20% resistance to cefepime (fourth-generation). Rates for tobramycin and amoxicillin-clavulanate were below 15%, whereas those for quinolones, gentamicin, sulfonamides, and penicillins ranged from 30 to 60% (Table 7).

**Table 7:** Antimicrobial resistance of *Escherichia coli* to key antimicrobial agents.

Antibacterial drugs	2016 *n* = 5	2017 *n* = 6	2018 *n* = 4	2019 *n* = 0	2020 *n* = 2	2021 *n* = 4	Total *n* = 21
Ciprofloxacin	80.00	60.00	50.00	ND*	100.00	25.00	57.84
Levofloxacin	80.00	66.67	50.00	ND*	50.00	50.00	61.90
Gentamycin	100.00	33.33	50.00	ND*	0.00	50.00	44.44
Tobramycin	0.00	0.00	25.00	ND*	0.00	0.00	6.67
Piperacillin	100.00	50.00	0.00	ND*	100.00	25.00	50.00
Ampicillin	100.00	40.00	50.00	ND*	100.00	100.00	68.75
Ampicillin-Sulbactam	ND*	0.00	0.00	ND*	100.00	50.00	33.33
Amoxicillin-Clavalanse	0.00	0.00	0.00	ND*	100.00	0.00	14.29
Aztreonam	60.00	16.67	50.00	ND*	0.00	25.00	33.33
Cefazolin	100.00	60.00	100.00	ND*	100.00	100.00	88.24
Cefuroxime	66.67	60.00	0.00	ND*	100.00	75.00	66.67
Cefoxitin	0.00	0.00	0.00	ND*	100.00	0.00	6.67
Ceftriaxone	20.00	20.00	50.00	ND*	50.00	50.00	35.00
Cefperazone-Sulbactam	0.00	0.00	25.00	ND*	0.00	0.00	7.69
Cefotaxime	75.00	33.33	ND*	ND*	ND*	ND*	57.14
Ceftazidime	75.00	0.00	0.00	ND*	0.00	0.00	18.75
Cefepime	40.00	0.00	25.00	ND*	0.00	25.00	19.05
Cotrimoxazole	100.00	40.00	50.00	ND*	0.00	50.00	50.00
Fosfomycin	ND*	ND*	ND*	ND*	100.00	ND*	100.00

*ND indicates that antimicrobial susceptibility testing for these agents was not performed.

### 3.6 Risk factor analysis of chronic osteomyelitis infection

#### 3.6.1 Univariate analysis of risk factors

Univariate analysis revealed that sex, age, BMI, alcohol consumption, and disease duration were not significantly associated with pathogenic infection in chronic osteomyelitis (*p* > 0.05). Conversely, a history of trauma, diabetes, hypertension, hospital stay, and smoking emerged as significant risk factors (*p* < 0.05) (Table 8).

**Table 8:** Univariate analysis of risk factors for pathogen infection in 421 cases of chronic osteomyelitis.

Items	Positive	Total	*P*–value
Age			
≤20	25 (8.56)	42 (9.98)	0.920
21–40	70 (23.97)	101 (23.99)	
41–60	118 (40.41)	170 (40.38)	
≥61	79 (27.05)	108 (25.65)	
Gender			
Male	224 (76.71)	317 (75.30)	0.311
Female	68 (23.29)	104 (24.70)	
BMI			
<18.5	11 (3.77)	20 (4.75)	0.228
18.5–23.9	105 (35.96)	156 (37.06)	
≥24	176 (60.27)	245 (58.19)	
Diabetes			
No	228 (78.08)	342 (81.24)	0.013
Yes	64 (21.92)	79 (18.76)	
Hypertension			
No	233 (79.79)	350 (83.14)	0.006
Yes	59 (20.21)	71 (16.86)	
Smoking history			
No	171 (58.56)	261 (62.00)	0.029
Yes	121 (41.44)	160 (38.00)	
Drinking history			
No	214 (73.29)	316 (75.06)	0.206
Yes	78 (26.71)	105 (24.94)	
Trauma history			
No	96 (32.88)	159 (37.77)	0.002
Yes	196 (67.12)	262 (62.23)	
Hospitalization time [days, median (IQR)]	20 (IQR 14–28)	18 (IQR 12–26)	0.000
Duration of illness [months, median (IQR)]	6 (IQR 2–21)	5 (IQR 2–17)	0.366

*Drinking history: consuming at least one standard drink (14 g of pure ethanol) per day for one consecutive year or more, or having abstained from alcohol for less than 1 year. Smoking history: Smoking at least one cigarette per day for one consecutive year or more, or having quit for less than 1 year. Trauma history: A history of previous trauma that required medical attention at a hospital.

#### 3.6.2 Binary logistic regression analysis of chronic osteomyelitis infection

We included variables found to be significant in the univariate analysis (trauma history, diabetes, hypertension, hospital stay, and smoking status) in the binary logistic regression analysis. The analysis revealed that hospitalization duration, diabetes, and smoking status were independent risk factors for chronic osteomyelitis (p < 0.05). Each additional hospital day increased the odds of infection by 3.7% (OR = 1.037, 95% CI 1.015–1.060, *p* = 0.001). Diabetic patients had 6.61-fold higher odds than non-diabetics (OR = 6.61, 95% CI 1.006–43.418, *p* = 0.049), and ever-smokers had 6.87-fold higher odds than never-smokers (OR = 6.873, 95% CI 1.930–24.479, *p* = 0.003) (Table 9).

**Table 9:** Logistic regression analysis of chronic osteomyelitis infection.

Items	Group	*Β*	Wald chi-square value	*p*-value	Odds ratio	95% CI
Hospitalization time		0.037	11.346	0.001	1.037	1.015–1.060
Diabetes	No					
	Yes	1.889	3.867	0.049	6.61	1.006–43.418
Smoking	No					
	Yes	1.928	8.848	0.003	6.873	1.93–24.479

## 4 Discussion

Osteomyelitis, a severe complication that often results from improper treatment of post-traumatic or post-surgical infections, becomes chronic when the condition persists for over a month, owing to incomplete treatment or low host resistance. Chronic osteomyelitis arises when acute infection persists beyond 1 month because of incomplete treatment or impaired host immunity. Incidence and prognosis vary geographically and temporally, reflecting differences in climate, lifestyle, cultural practices, healthcare quality and patient adherence; therefore, region-specific epidemiological data are essential for guiding local treatment strategies.

We enrolled 632 patients from four centers. The median age was 46 (IQR 30–58) years; 39.4% were 41–60 years old and 22.9% were >60 years old, aligning with Kremers et al. (9) and Ma et al. (14). Chronic osteomyelitis therefore predominantly affects middle-aged and older adults, a pattern that may reflect age-related immunosenescence and impaired wound healing. Overall, the male-to-female ratio was 2.49:1, peaking at 3.18:1 among 21–40 year olds and mirroring the 3.53:1 ratio reported by Jiang et al. (12). This male predominance likely reflects greater occupational and traffic-related trauma exposure. Lower-limb infections predominated (81.8%), particularly in the tibia (38.1%), consistent with earlier studies (12, 13, 15, 16). The tibia’s limited soft-tissue envelope and tenuous blood supply facilitate pathogen persistence and thus increase osteomyelitis risk (19).

Chronic osteomyelitis associated with bacterial infections triggers inflammatory responses. WBC, ESR, CRP, and PCT levels are key indicators of inflammation and are crucial for assessing chronic osteomyelitis. In our study, positivity rates were 15.2% for WBC, 45.4% for CRP, and 57.7% for ESR—values comparable to those reported by Lu et al. (16) and Huang et al. (17). Infection was considered present only when all three indicators (WBC, CRP, and ESR) were elevated. However, the probability of infection was 19.6% despite all indicators being negative (20). Michail et al. (21) observed that in patients with diabetic foot osteomyelitis, PCT, WBC, CRP, and ESR levels were elevated; however, after 7 days of antibiotic therapy, PCT, WBC, and CRP levels returned to normal ranges. Lavery et al. (22) observed that among patients with high ESR, concomitant CRP measurement improved discrimination between osteomyelitis and soft-tissue infection.

In this study, 386 pathogens were isolated from 421 specimens subjected to bacterial culture, corresponding to a positivity rate of 69.36%. This figure is comparable to the 66% reported by Sheehy et al. (23) and 67.7% by Dudareva et al. (24), but lower than the 85% of Ferreira et al. (25) and 93% of Mthethwa and Marais et al. (26). The discrepancies in positivity rates across these studies can be attributed to the uneven distribution of medical resources and varying levels of healthcare quality. Regions with advanced medical care have more standardized and precise methods for pathogen cultivation and detection, which may result in higher positivity rates. Furthermore, the differential use of antibiotics in these regions is important. The inappropriate use of antibiotics can cause increased bacterial resistance, a rise in drug-resistant strains, and a lower positivity rate in pathogen cultures. Gram-positive organisms predominated (63.2%), led by *S. aureus* (34.2%), followed by CoNS (12.2%). Among Gram-negative isolates, *Enterobacter* spp. (6.5%) and *Pseudomonas* spp. (6.2%) were most common. The share of *S. aureus* declined from 35.6% in 2016 to 29.0% in 2021, underscoring the dynamic nature of the pathogen spectrum. Continuous surveillance is therefore required to guide empirical therapy. Within their respective groups, *S. aureus* accounted for 54.1% of Gram-positive isolates, whereas *Enterobacter* spp. represented 18.0% of Gram-negative isolates—information that supports prompt antimicrobial selection at admission.

Antibiotics are widely used to treat chronic osteomyelitis; however, there is a rise in antibiotic resistance. In this study, multidrug-resistant bacteria were detected in 44.04% of cases, which was lower than in Dudareva’s study (24). Resistance is driven largely by biofilm formation. These extracellular polymeric matrices coat necrotic bone and implants, sheltering pathogens and potentiating antibiotic tolerance. Biofilms also suppress host immunity by impeding neutrophil respiration, promoting bacterial dissemination and enabling inter-cellular signaling, ultimately leading to persistent infection, recurrent lesions and reduced quality of life (27, 28).

Antimicrobial susceptibility testing showed high resistance to standard agents in both Gram-positive and Gram-negative isolates. *Staphylococci* (MSSA, MRSA, and CoNS) resistance to penicillin was >85%, extending to 100% and 50–90% resistance to erythromycin. *Enterococci* exhibited >75% resistance to erythromycin and clindamycin. However, Gram-positive bacteria demonstrated <10% resistance to glycopeptides and oxazolidinones, with some showing complete susceptibility. MSSA is sensitive to tetracycline, rifampicin, and ciprofloxacin; however, careful consideration is required due to the liver toxicity of rifampicin and the neurological and cardiac toxicities of quinolones. Vancomycin, linezolid, and teicoplanin were highly effective for drug-resistant Gram-positive infections. Gram-negative bacteria showed higher resistance to the first and second generation cephalosporins than to the third- and fourth-generation. For example, 86% of *Enterobacter* species were resistant to cefazolin, a first generation cephalosporin; however, only 8% were resistant to cefepime, a fourth generation cephalosporin. *Pseudomonas* species had 100% resistance to first- and second-generation cephalosporins and 4% resistance to third and fourth generation cephalosporins. In addition, ceftazidime-avibactam were effective against *Pseudomonas aeruginosa* with low resistance rates.

Binary logistic regression identified diabetes, prolonged hospitalization and smoking as independent risk factors for chronic osteomyelitis, in line with earlier reports (29–32). Diabetic patients carried a 6.6-fold higher infection risk than non-diabetics, probably because chronic hyperglycaemia provides a nutrient-rich milieu for bacteria (33) and because diabetes-associated microangiopathy impairs bone perfusion, weakening local defence and repair (34). Each additional hospital day increases the odds of infection by 3.7%. Prolonged hospitalization in osteomyelitis patients reflects greater disease severity and indicates a complex clinical course requiring ongoing evaluation and treatment. Smokers are 6.873 times more at risk of infection than non-smokers. Nicotine-induced vasoconstriction compromises bone blood flow (35), while smoke exposure impairs immunity (35, 36) and promotes bacterial adhesion and biofilm formation on implants—phenotypes linked to persistent, drug-resistant infection and chronic osteomyelitis (37).

Polymicrobial infections increase the failure rate of empirical therapy and accelerate the emergence of antibiotic resistance (38, 39). A likely mechanism is metabolic complementarity among mixed flora, which locally alters pH, oxygen tension, and antibiotic concentrations, creating sub-inhibitory zones that foster and enrich resistance mutations (39–41). Compared with single-strain isolates, mixed-strain populations show a higher rise in MIC under identical β-lactam pressure (42). Therefore, mixed infections should be managed with anti-biofilm combination regimens, and multi-site specimens should be sent promptly for whole-genome sequencing to monitor resistance evolution in real time.

This study has several limitations. First, TO, HO, and DO may differ in pathophysiology, microbial profiles, risk factors, and therapeutic approaches. Owing to the constraints of the current dataset, this study focused on presenting the overall regional pathogen spectrum and resistance patterns to provide baseline data for empirical therapy. Prospective studies are warranted to delineate the pathogen distribution, resistance patterns, and risk factors specific to each osteomyelitis subtype. Second, as a retrospective study, preadmission antibiotic use data could not be obtained, which might have affected pathogen detection and laboratory results. Larger prospective studies are needed to provide more robust evidence for prevention and treatment.

In our study, chronic osteomyelitis was most prevalent among men aged 41–60 years, with post-traumatic disease predominating. Infections centered on the lower limb–especially the tibia, followed by the femur and calcaneus. Gram-positive bacteria–especially *S. aureus* and CoNS–together with Gram-negative pathogens such as *Enterobacter* spp. and *Pseudomonas* spp., are the primary agents implicated in chronic osteomyelitis. *S. aureus* prevalence is declining; however, MRSA and CoNS rates are on the rise. Gram-positive bacteria are highly resistant to conventional antibiotics such as penicillin and erythromycin; nevertheless, they remain sensitive to linezolid and vancomycin. Third and fourth-generation cephalosporins (e.g., cefepime) and carbapenems (e.g., meropenem) are recommended for Gram-negative infections. Longer hospital stay, current smoking and diabetes were independent risk factors; their effective control is essential for recovery.

## Data Availability

The original contributions presented in the study are included in the article/supplementary material, further inquiries can be directed to the corresponding authors.

## References

[1] Panteli M Giannoudis PV. Chronic osteomyelitis: what the surgeon needs to know. EFORT Open Rev. (2016) 1:128–35. doi: 10.1302/2058-5241.1.000017, PMID: 28461939 PMC5367612

[2] Heitzmann LG Battisti R Rodrigues AF Lestingi JV Cavazzana C Queiroz RD. Postoperative chronic osteomyelitis in the long bones-current knowledge and Management of the Problem. Rev Bras Ortop. (2019) 54:627–35. doi: 10.1016/j.rbo.2017.12.013, PMID: 31875060 PMC6923639

[3] Rafferty BA Thakrar P. Chronic recurrent multifocal osteomyelitis. Med Clin North Am. (2024) 108:227–39. doi: 10.1016/j.mcna.2023.05.022, PMID: 37951653

[4] Senneville EM Lipsky BA van Asten SAV Peters EJ. Diagnosing diabetic foot osteomyelitis. Diabetes Metab Res Rev. (2020) 36:e3250. doi: 10.1002/dmrr.3250 31950555

[5] Moreno-Mateo F Perea SH Onel KB. Chronic recurrent multifocal osteomyelitis: diagnosis and treatment. Curr Opin Pediatr. (2021) 33:90–6. doi: 10.1097/MOP.0000000000000970, PMID: 33278106

[6] Dartnell J Ramachandran M Katchburian M. Haematogenous acute and subacute paediatric osteomyelitis: a systematic review of the literature. J Bone Joint Surg Br. (2012) 94:584–95. doi: 10.1302/0301-620X.94B5.28523, PMID: 22529075

[7] Kim J Lee MU Kim T-H. Nationwide epidemiologic study for pediatric osteomyelitis and septic arthritis in South Korea: a cross-sectional study of national health insurance review and assessment service. Medicine. (2019) 98:e15355. doi: 10.1097/MD.0000000000015355, PMID: 31027117 PMC6831362

[8] Walter N Baertl S Alt V Rupp M. What is the burden of osteomyelitis in Germany? An analysis of inpatient data from 2008 through 2018. BMC Infect Dis. (2021) 21:550. doi: 10.1186/s12879-021-06274-6, PMID: 34112102 PMC8194128

[9] Kremers HM Nwojo ME Ransom JE Wood-Wentz CM Melton LJ Huddleston PM. Trends in the epidemiology of osteomyelitis: a population-based study, 1969 to 2009. J Bone Joint Surg Am. (2015) 97:837–45. doi: 10.2106/JBJS.N.01350, PMID: 25995495 PMC4642868

[10] Ren Y Liu L Sun D Zhang Z Li M Lan X. Epidemiological updates of post-traumatic related limb osteomyelitis in China: a 10 years multicentre cohort study. Int J Surg. (2023) 109:2721–31. doi: 10.1097/JS9.0000000000000502 37247014 PMC10498838

[11] Lowenberg DW DeBaun M Suh GA. Newer perspectives in the treatment of chronic osteomyelitis: a preliminary outcome report. Injury. (2019) 50:S56–61. doi: 10.1016/j.injury.2019.04.016, PMID: 31079834

[12] Jiang N Ma Y-F Jiang Y Zhao X-Q Xie G-P Hu Y-J. Clinical characteristics and treatment of extremity chronic osteomyelitis in southern China: a retrospective analysis of 394 consecutive patients. Medicine. (2015) 94:e1874. doi: 10.1097/MD.0000000000001874, PMID: 26496345 PMC4620766

[13] Wang X Yu S Sun D Fu J Wang S Huang K. Current data on extremities chronic osteomyelitis in Southwest China: epidemiology, microbiology and therapeutic consequences. Sci Rep. (2017) 7:16251. doi: 10.1038/s41598-017-16337-x, PMID: 29176616 PMC5701171

[14] Ma X Han S Ma J Chen X Bai W Yan W. Epidemiology, microbiology and therapeutic consequences of chronic osteomyelitis in northern China: a retrospective analysis of 255 patients. Sci Rep. (2018) 8:14895. doi: 10.1038/s41598-018-33106-6, PMID: 30291260 PMC6173749

[15] Zhang X Lu Q Liu T Li Z Cai W. Bacterial resistance trends among intraoperative bone culture of chronic osteomyelitis in an affiliated hospital of South China for twelve years. BMC Infect Dis. (2019) 19:823. doi: 10.1186/s12879-019-4460-y, PMID: 31533647 PMC6751654

[16] Lu S Wang L Luo W Wang G Zhu Z Liu Y. Analysis of the epidemiological status, microbiology, treatment methods and financial burden of hematogenous osteomyelitis based on 259 patients in Northwest China. Front Endocrinol. (2022) 13:1097147. doi: 10.3389/fendo.2022.1097147, PMID: 36686458 PMC9846127

[17] Huang X Li Q Chen J Liu T Zhao Y Teng Y. Clinical features of chronic tibial osteomyelitis: a single-center retrospective study of 282 cases in Xinjiang, China. BMC Musculoskelet Disord. (2024) 25:823. doi: 10.1186/s12891-024-07928-7, PMID: 39427137 PMC11490011

[18] Magiorakos AP Srinivasan A Carey RB Carmeli Y Falagas ME Giske CG. Multidrug-resistant, extensively drug-resistant and pandrug-resistant bacteria: an international expert proposal for interim standard definitions for acquired resistance. Clin Microbiol Infect. (2012) 18:268–81. doi: 10.1111/j.1469-0691.2011.03570.x, PMID: 21793988

[19] Wijendra A Ramsden A McNally M. A natural history of untreated chronic osteomyelitis of the tibia over 20 years, with evolving squamous cell carcinoma: a case report. J Bone Jt Infect. (2023) 8:183–8. doi: 10.5194/jbji-8-183-2023, PMID: 37780529 PMC10539787

[20] Stucken C Olszewski DC Creevy WR Murakami AM Tornetta P. Preoperative diagnosis of infection in patients with nonunions. J Bone Joint Surg Am. (2013) 95:1409–12. doi: 10.2106/JBJS.L.01034, PMID: 23925746

[21] Michail M Jude E Liaskos C Karamagiolis S Makrilakis K Dimitroulis D. The performance of serum inflammatory markers for the diagnosis and follow-up of patients with osteomyelitis. Int J Low Extrem Wounds. (2013) 12:94–9. doi: 10.1177/1534734613486152, PMID: 23667102

[22] Lavery LA Ahn J Ryan EC Bhavan K Oz OK La Fontaine J. What are the optimal cutoff values for ESR and CRP to diagnose osteomyelitis in patients with diabetes-related foot infections? Clin Orthop Relat Res. (2019) 477:1594–602. doi: 10.1097/CORR.0000000000000718, PMID: 31268423 PMC6999976

[23] Sheehy SH Atkins BA Bejon P Byren I Wyllie D Athanasou NA. The microbiology of chronic osteomyelitis: prevalence of resistance to common empirical anti-microbial regimens. J Infect. (2010) 60:338–43. doi: 10.1016/j.jinf.2010.03.006, PMID: 20230854

[24] Dudareva M Hotchen AJ Ferguson J Hodgson S Scarborough M Atkins BL. The microbiology of chronic osteomyelitis: changes over ten years. J Infect. (2019) 79:189–98. doi: 10.1016/j.jinf.2019.07.006, PMID: 31319142

[25] Ferreira N Reddy K Venter RG Centner CM Laubscher M. Antibiogram profiles and efficacy of antibiotic regimens of bacterial isolates from chronic osteomyelitis of the appendicular skeleton: a developing-world perspective. S Afr Med J. (2021) 111:642–8. doi: 10.7196/SAMJ.2021.v111i7.15516, PMID: 34382547

[26] Mthethwa PG Marais LC. The microbiology of chronic osteomyelitis in a developing world setting. SA Orthop J. (2017) 16:39–45. doi: 10.17159/2309-8309/2017/v16n2a4

[27] Taylor PK Yeung ATY Hancock REW. Antibiotic resistance in *Pseudomonas aeruginosa* biofilms: towards the development of novel anti-biofilm therapies. J Biotechnol. (2014) 191:121–30. doi: 10.1016/j.jbiotec.2014.09.003, PMID: 25240440

[28] Grooters KE Ku JC Richter DM Krinock MJ Minor A Li P. Strategies for combating antibiotic resistance in bacterial biofilms. Front Cell Infect Microbiol. (2024) 14:1352273. doi: 10.3389/fcimb.2024.1352273, PMID: 38322672 PMC10846525

[29] Lavery LA Peters EJG Armstrong DG Wendel CS Murdoch DP Lipsky BA. Risk factors for developing osteomyelitis in patients with diabetic foot wounds. Diabetes Res Clin Pract. (2009) 83:347–52. doi: 10.1016/j.diabres.2008.11.030, PMID: 19117631

[30] Yalikun A Yushan M Li W Abulaiti A Yusufu A. Risk factors associated with infection recurrence of posttraumatic osteomyelitis treated with Ilizarov bone transport technique-a retrospective study of 149 cases. BMC Musculoskelet Disord. (2021) 22:573. doi: 10.1186/s12891-021-04430-2, PMID: 34162362 PMC8223287

[31] Slyamova G Gusmanov A Batpenov A Kaliev N Viderman D. Risk factors for postoperative osteomyelitis among patients after bone fracture: a matched case-control study. J Clin Med. (2022) 11:6072. doi: 10.3390/jcm11206072, PMID: 36294391 PMC9604902

[32] Zhou X Ma S Xu Y Sun C Liao J Song M. Nicotine promotes *Staphylococcus aureus*-induced osteomyelitis by activating the Nrf2/Slc7a11 signaling axis. Int Immunopharmacol. (2024) 135:112223. doi: 10.1016/j.intimp.2024.112223, PMID: 38772295

[33] Chávez-Reyes J Escárcega-González CE Chavira-Suárez E León-Buitimea A Vázquez-León P Morones-Ramírez JR. Susceptibility for some infectious diseases in patients with diabetes: the key role of Glycemia. Front Public Health. (2021) 9:559595. doi: 10.3389/fpubh.2021.559595, PMID: 33665182 PMC7921169

[34] Sharma P Sharma RK Gaur K. Understanding the impact of diabetes on bone health: a clinical review. Metabol Open. (2024) 24:100330. doi: 10.1016/j.metop.2024.100330, PMID: 39606009 PMC11600011

[35] Tarantino U Cariati I Greggi C Gasbarra E Belluati A Ciolli L. Skeletal system biology and smoke damage: from basic science to medical clinic. Int J Mol Sci. (2021) 22:6629. doi: 10.3390/ijms22126629, PMID: 34205688 PMC8234270

[36] Qiu F Liang C-L Liu H Zeng Y-Q Hou S Huang S. Impacts of cigarette smoking on immune responsiveness: up and down or upside down? Oncotarget. (2017) 8:268–84. doi: 10.18632/oncotarget.13613, PMID: 27902485 PMC5352117

[37] Hu R Qian H Wang X Peng B Huang D. Nicotine promotes pathogenic bacterial growth and biofilm formation in peri-implant. J Med Microbiol. (2024) 73:1897. doi: 10.1099/jmm.0.001897, PMID: 39360709

[38] Wu M Guo F He X Zheng D Ye W Li S. Analysis of distribution and drug susceptibility test results of pathogenic bacteria in diabetic foot ulcers. Diabetes Ther. (2024) 15:1627–37. doi: 10.1007/s13300-024-01601-x, PMID: 38771473 PMC11211311

[39] Uddin TM Chakraborty AJ Khusro A Zidan BRM Mitra S Emran TB. Antibiotic resistance in microbes: history, mechanisms, therapeutic strategies and future prospects. J Infect Public Health. (2021) 14:1750–66. doi: 10.1016/j.jiph.2021.10.020, PMID: 34756812

[40] Campey A Łapińska U Chait R Tsaneva-Atanasova K Pagliara S. Antibiotic resistant bacteria survive treatment by doubling while shrinking. MBio. (2024) 15:e0237524. doi: 10.1128/mbio.02375-24, PMID: 39565111 PMC11633386

[41] Ahmad M Aduru SV Smith RP Zhao Z Lopatkin AJ. The role of bacterial metabolism in antimicrobial resistance. Nat Rev Microbiol. (2025) 23:439–54. doi: 10.1038/s41579-025-01155-0, PMID: 39979446 PMC12173792

[42] Daruka L Czikkely MS Szili P Farkas Z Balogh D Grézal G. ESKAPE pathogens rapidly develop resistance against antibiotics in development in vitro. Nat Microbiol. (2025) 10:313–31. doi: 10.1038/s41564-024-01891-8, PMID: 39805953 PMC11790497

